# Early Cardiac Ischemia–Reperfusion Injury: Interactions of Autophagy with Galectin-3 and Oxidative Stress

**DOI:** 10.3390/biomedicines12112474

**Published:** 2024-10-28

**Authors:** Suhail Al-Salam, Satwat Hashmi, Govindan S. Jagadeesh, Manjusha Sudhadevi, Aktham Awwad, Abderrahim Nemmar

**Affiliations:** 1 Department of Pathology, College of Medicine and Health Sciences, United Arab Emirates University, Al Ain P.O. Box 15551, United Arab Emirates; jagadeesh.sangaran@uaeu.ac.ae (G.S.J.); m.sudhadevi@uaeu.ac.ae (M.S.); 2 Department of Biological and Biomedical Sciences, Agha Khan University, Karachi City 74000, Pakistan; satwat.hashmi@aku.edu; 3 Department of Laboratory Medicine, Tawam Hospital, Al Ain P.O. Box 5674, United Arab Emirates; aktham.awwad@purelab.com; 4 Department of Physiology, College of Medicine and Health Sciences, United Arab Emirates University, Al Ain P.O. Box 15551, United Arab Emirates; anemmar@uaeu.ac.ae

**Keywords:** heart, myocardial ischemia–reperfusion, galectin-3, autophagy, oxidative stress

## Abstract

**Background**: Cardiovascular diseases are the leading cause of death worldwide, including the United Arab Emirates. Ischemia–reperfusion (IR) injury results in the death of cardiac myocytes that were viable immediately before myocardial reperfusion. We aim to investigate the role of galectin-3 (Gal-3) in autophagy during ischemia–reperfusion injuries. **Methods**: Male C57B6/J and Gal-3 knockout (KO) mice were used for the murine model of IR injury. Heart samples and serum were collected 24 h post-IR and were processed for immunohistochemical and immunofluorescent labeling and an enzyme-linked immunosorbent assay. **Results**: There was a significant increase in left ventricle (LV) concentrations of Gal-3 in Gal-3 wild-type mice compared to sham mice. There were significantly higher concentrations of LV autophagy proteins and phospho-AMPK in IR Gal-3 KO mice than in IR Gal-3 wild-type mice, compared to lower concentrations of LV phospho-mTOR and p62 in IR Gal-3 KO than in IR wild-type mice. Antioxidant activities were higher in the LVs of IR Gal-3 wild-type mice, while oxidative stress was higher in the LVs of IR Gal-3 KO mice. **Conclusions**: Our study supports the interaction of Gal-3 with autophagy proteins, oxidative stress, and antioxidant proteins and demonstrates that the absence of Gal-3 can enhance autophagy in the heart after IR injury.

## 1. Introduction

Timely and effective restoration of blood flow after a heart attack (MI) is the best approach for preserving heart muscle and enhancing recovery outcomes. However, while this process brings oxygen back to the affected heart tissue, it can also cause reperfusion injury, potentially diminishing the positive impact of the treatment. Ischemia–reperfusion (IR) injury leads to the death of cardiomyocytes that were still viable before the blood flow was restored [1].

Autophagy is an intracellular process that deals with lysosomal degradation of damaged proteins and micro-organelles and allows recycling of the constituents to be used for many cellular activities [2]. There is evidence supporting that autophagy plays a protective role by maintaining protein quality control and cellular homeostasis. However, excessive autophagy can lead to cell death [2].

In the heart, autophagy contributes to cellular homeostasis through the degradation of long-lived or surplus proteins and aged organelles. Sai Ma et al. showed that the deletion of autophagy genes can lead to serious effects on the myocardium, like impaired contractile function leading to cardiomyopathy and heart failure [3]. Altered autophagy processes have been observed in ischemic heart disease (IHD), such as the impairment of autophagic flux leading to the accumulation of damaged organelles and proteins [4]. Autophagic flux refers to the whole process of autophagy, from the formation of autophagosomes to their fusion with lysosomes and degradation of the cargo [5]. Early in the course of IHD, there may be an increase in the formation of autophagosomes; however, if this increase is not matched by adequate fusion with lysosomes and degradation, it can lead to autophagosome accumulation and increased cellular stress, leading to cardiomyocyte death [5,6].

Autophagy is activated during IR injury to increase energy production, re-cycling damaged proteins and organelles and clearing intracellular toxic substances [7]. During myocardial ischemia, there is a low ATP level within cardiomyocytes, leading to the upregulation of Adenosine monophosphate-activated protein kinase (AMPK), which inactivates mTOR, leading to increased autophagy and increased ATP production [7]. During reperfusion, there is inactivation of AMPK, and the autophagy will be initiated by the upregulation of Beclin1 [8]. Oxygen free radicals that are generated during the perfusion phase of IR injury is the major factor in Beclin1 upregulation [5,9].

Hariharan N et al. showed that antioxidant treatment can protect cardiomyocytes against IR injury, which is accompanied by the suppression of autophagic flux, which may be protective during ischemia/reperfusion [9].

In autophagy, the process of degradation is relatively non-specific in nature, which may lead to the digestion of organelles and proteins that have protective effects on cardiomyocytes during IR injury [10]. Scherz-Shouval R et al. showed that autophagy can degrade catalase, a critical enzyme reducing H_2_O_2_, during IR injury [10], leading to increase in oxidative stress and programed cell death. Decker RS et al. also showed that there is a direct correlation between the severity of ischemia and the extent of autophagy during the reperfusion phase [11]. When ischemia is mild, the activation of autophagy during reperfusion is mild and may not be harmful [12]. In addition, the inhibition of autophagy at the level of autophagosome formation and at the level of autophagosome–lysosome fusion may differentially affect ischemia/reperfusion injury [12].

Gal-3, a 35 kDa protein, is present on the surface of cells, within the extracellular matrix, and inside the cytoplasm and nucleus, and the location varies depending on factors like the type of cell and its proliferation state [13,14,15,16,17,18], cultivation conditions [19], neoplastic progression [20,21,22,23,24], and transformation [25]. The distribution in many types of cells, together with varied subcellular localization, indicates that Gal-3 has many different roles in normal and pathophysiological conditions [26,27].

Oka, N et al. [28], showed that intracellular Gal-3 plays a role in cell division, growth, and mechanisms that prevent apoptosis. It influences K-Ras and Akt proteins, thereby controlling processes like cell differentiation, survival, and programmed cell death [23,28]. Extracellular Gal-3 is involved in mediating interactions between cells, signaling pathways, cell–matrix binding, apoptosis, and inflammation [29].

Studies showed that Gal-3 can down regulate autophagy proteins, LC3B and p62, in melanoma and endothelial cell lines [30,31]. It was also shown that Gal-3 can regulate the AMPK-mTOR signaling pathway in endothelial cell lines, leading to the upregulation of AMPK and downregulation of mTOR [31]. Weng et al. recognized that the intracellular accumulation of Gal-3 triggers a significant anti-autophagic reaction, which occurs as Gal-3 detects alterations in glycosylation on the cell surface and adjusts cellular responses by recognizing different glycans present on damaged phagosomal membranes [32]. Gal-3 protects cells from lysosomal cell death or microbial invasion through its interaction with its partner Tripartite motif-containing protein 16 (TRIM16), which serves as a scaffold protein by interacting with autophagic proteins p62, ULK1, ATG16L1, and LC3B, facilitates the autophagic degradation of protein aggregates and streamlines the process of stress-induced aggregate clearance and protects cells against oxidative stress-induced toxicity [33,34]. This study investigated the role of Gal-3 in autophagy during early IR injury. 

## 2. Materials and Methods

### 2.1. Mouse Model of Myocardial Ischemia–Reperfusion

Mice were anesthetized with an intraperitoneal injection made with a combination of Ketamine (100 mg/kg) and Xylazine (10 mg/kg) and fixed on the operating pad in the supine position by tapping all four extremities. The animals were then intubated and ventilated (Harvard apparatus Minivent Hugo Sachs Electronik, Holliston, MA, USA). The experiment was performed under a stereomicroscope (ZEISS, Oberkochen, Germany. The ventilator was connected to an oxygen source providing adequate oxygen throughout the experiment. The chest was opened with a lateral cut of the 4th intercostal space on the left side of the sternum. Next, the chest walls were retracted for better visualization of the heart. With minimal manipulation, the pericardial sac was removed, and the left atrial auricle retracted to visualize the left anterior descending artery (LAD). An 8-0 silk suture was passed under the LAD at 1 mm distal to the left atrial appendage. For the myocardial reperfusion model, a PE tube was put under the suture and knotted on top of the tube to temporarily occlude the LAD. It was doubly ligated, and the disruption of blood flow was visualized by discoloration of the left ventricle. An accompanying ECG recording showed characteristic ST-Elevation (AD Instruments, Sydney, Australia), which further confirmed ischemia. After 30 min of ischemia, the knot was cut by micro lancet, and reperfusion was observed. The chest wall was closed by bringing the third and fourth ribs together using one or two interrupted sutures. The muscles were repositioned, and the skin was closed with a continuous 6-0 prolene suture. The mouse was carefully removed from the ventilator, and spontaneous breathing resumed immediately.

All experimental animal procedures conformed to the rules and regulation laid down by the Animals Research Ethics Committee of the UAEU, Protocol ERA_2023_2807.

### 2.2. Mice Strains

In this experiment, 16 wild-type (WT) C57B6/J mice and 16 GAL-3 knockout (KO) mice (The Jackson Laboratory, 006338, galactin3-, B6 Cg-Lgals3 <tm 1 Poi>/J, homozygous genotype) were used. All animals were male, between 12 and 16 weeks old, and weighed 20–25 g. Sham-operated mice (n = 16) from each group were also included in this study. In each group, 8 hearts were collected for protein analysis, while the remaining 8 hearts were used for histological analysis.

### 2.3. Troponin-I Assay

Mouse cardiac troponin I levels in plasma were measured by using a high sensitivity mouse cardiac troponin-I Elisa kit (2010-1-HSP, Life Diagnostics, Inc., West Chester, PA, USA) according to the manufacturer’s instructions.

### 2.4. Immunohistochemistry and Immunofluorescence Labeling

This project analyzed the immunohistochemical autophagic profile of cardiomyocytes that are injured by ischemia and subsequent reperfusion to identify the site of expression of Gal-3 and autophagy proteins. For immunohistochemical analysis, five-micrometer sections were prepared and mounted on aminopropyltriethoxysilane (APES) (Sigma-Aldrich, St. Louis, MO, USA) coated slides. After dewaxing with xylene and rehydrating with graded alcohol, sections were then placed in an EnVisionTM FLEX Target Retrieval Solution with a high PH (PH 9) (DAKO Agilent, Santa Clara, CA, USA) in a water bath at 95 °C for 30 min. Sections were then treated with peroxidase block. All the primary antibodies (anti-p62, anti-LC3-B, anti-anti-phospho-mTOR (p-mTOR), anti-phospho-AMPK (p-AMPK), anti-catalase, and anti-NRF2 (Rabbit monoclonal, Cell Signaling Technology, Danvers, MA, USA) of our interest were applied on sections with the appropriate concentration for one hour at room temperature, followed by washing in phosphate-buffered saline (PBS) for 15 min in three changes. The visualization of reaction was performed by EnVision Detection Systems (DAKO, Agilent, Santa Clara, CA, USA) with the Peroxidase/DAB technique (DAKO, Agilent, Santa Clara, CA, USA), which was followed by washing and counter staining with haematoxylin. Sections were then dehydrated, cleared, and mounted in DPX. Appropriate positive controls were used. For negative control, the primary antibody was not added to the section, and the whole procedure proceeded in this way.

For immunofluorescence labeling, 5-μm sections were deparaffinized with xylene and rehydrated with graded alcohol. Sections were placed in a Target Retrieval Solution with a high PH (PH 9) (DAKO, Agilent, Santa Clara, CA, USA), in a water bath at 95 °C for 30 min. Sections were later incubated with anti-galectin-3 (Santa Cruz Biotechnology, Dallas, TX, USA) overnight at room temperature. Sections were subsequently incubated with Donkey anti-rabbit Ig conjugated-anti-rabbit Alexa Fluor 488 antibodies (Jackson Laboratory, Bar Harbor, ME, USA). Finally, sections were mounted with DAPI-water-soluble mounting media (Abcam, Cambridge, MA, USA) and observed using an Olympus Fluorescent microscope (Olympus, Hamburg, Germany). Positive and negative controls were used in every batch of slides that were stained.

### 2.5. Morphometric Analysis

Morphometric analysis was conducted on stained sections with LC3B, p62, p-AMPK, mTOR, catalase and NRF2 expression in LV cells using ImageJ software (https://imagej.net/ij/ (1.53t version, National Health Institute, Bethesda, MD, USA). LC3B, p62, p-AMPK, mTOR, catalase, and NRF2 labeling were assessed by counting the number of positive cells in randomly selected high-power fields (HPFs) within the left ventricle (LV). The average number of positive cells was then converted from per HPF to per mm^2^ (with 1 mm^2^ equaling 4 HPF). For LC3B, p62, p-AMPK, mTOR, and catalase, cells were considered positive if they exhibited cytoplasmic staining, while for NRF2 labeling, cells were counted as positive if they showed nuclear staining.

### 2.6. Protein Extraction

Total protein was extracted from heart samples by homogenizing with lysis buffer and collecting the supernatant after centrifugation. Total protein concentration was determined by the BCA protein assay method.

### 2.7. Enzyme-Linked Immunosorbent Assay

Left ventricular myocardial concentration of Gal-3 was measured using the DuoSet enzyme-linked immunosorbent assay (ELISA) development kit from R&D Systems (Minneapolis, MN, USA). Meanwhile, Beclin1, p62, LC3-B, ATG5, p-mTOR, p-AMPK, ATG12, ATG13, ULK1, ATG9A, NRF2) concentrations were determined using MyBioSource ELISA kits (San Diego, CA, USA) and the standard procedure according to the manufacturer’s instructions.

This technique determines the levels of Gal-3, Beclin1, p62, LC3-B, ATG5, p-mTOR, p-AMPK, ATG12, ATG13, ULK1, ATG9A, NRF2 during IR injury as well as the changes in autophagy protein levels according to the presence or absence of Gal-3.

### 2.8. Oxidative Stress Markers and Antioxidant Enzyme Status Measurements

8-isoprosten concentration in the LV was measured using the Cayman ELISA kit (Cayman Chemical, Ann Arbor, MI 48108, USA). LV H_2_O_2_ levels were measured using the Colorimetric Assay Kit (Elabscience, Houston, TX 77079, USA). LV antioxidant enzyme levels of superoxide dismutase, Glutathione, and Catalase activity were measured by the Cayman ELISA kit (Cayman Chemical, Ann Arbor, MI 48108, USA). This step determines whether the modulation of Gal-3 and autophagy proteins have effects on the levels of oxidative stress and antioxidants in the LV during IR injury and vice versa.

### 2.9. Statistical Analysis

All statistical analyses were performed using GraphPad Prism software (version 5). Multiple group comparisons were conducted using a one-way analysis of variance (ANOVA), followed by Newman–Keuls post hoc multiple range tests. Comparisons between two groups were analyzed using a Student’s *t*-test. Data are presented as mean ± standard error (S.E.), and statistical significance is defined as *p* < 0.05.

## 3. Results

### 3.1. Troponin I Is Increased at 24 h Following IR Injury

**Figure 1:**
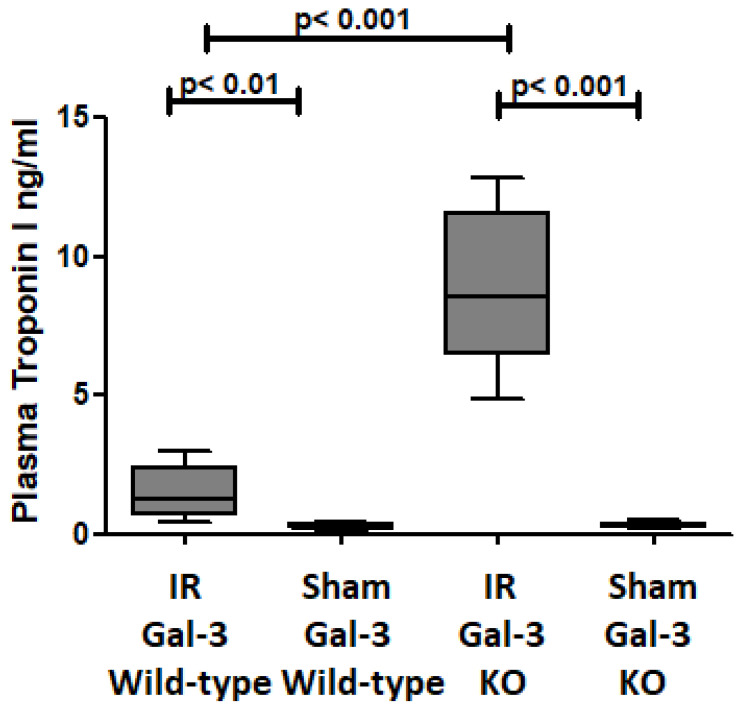
The graph illustrates plasma troponin I levels after IR injury in IR Gal-3 wild-type mice and IR Gal-3 KO mice, compared to their respective sham controls.

Plasma Troponin I concentration was significantly increased in the IR Gal-3 wild-type mice (1.513 ± 0.4043 pg/mg protein) compared to their sham control (0.2872 ± 0.04157 pg/mg protein) *p* < 0.01 (Figure 1), confirming cardiomyocyte death during IR injury. Plasma Troponin I concentration was significantly increased in the IR Gal-3 KO mice (8.811 ± 1.193 pg/mg protein) compared to their sham control (0.3393 ± 0.04280 pg/mg protein) *p* < 0.001 (Figure 1), confirming cardiomyocyte death during IR injury. Plasma Troponin I concentration was significantly higher in the IR Gal-3 KO mice than in the IR Gal-3 wild-type mice *p* < 0.001 (Figure 1).

### 3.2. Gal-3 Is Increased After Ischemia–Reperfusion Injury in the Left Ventricle

**Figure 2:**
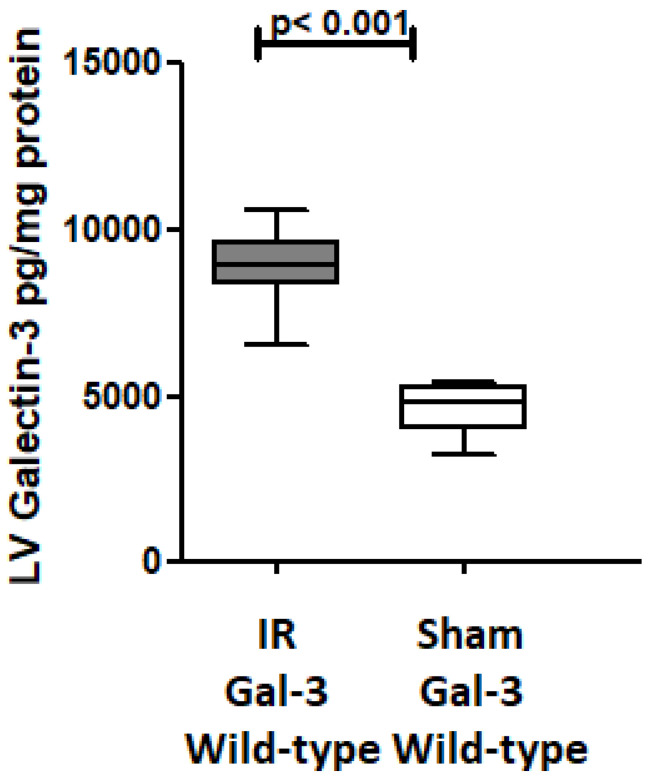
The graph depicts LV GAL-3 concentrations following IR in IR Gal-3 wild-type mice compared to their sham controls.

**Figure 3:**
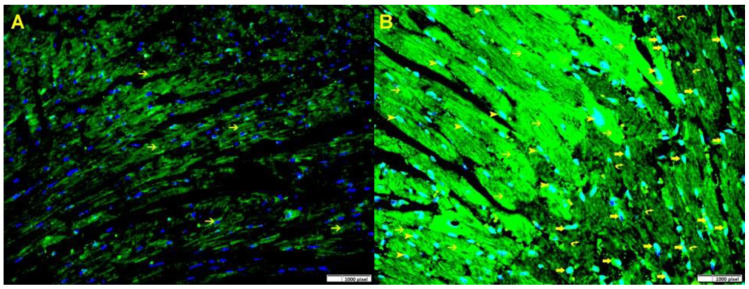
(**A**). A representative section of the LV from Gal-3 wild-type sham control mice displays Gal-3 expression in cardiac myocytes (thin arrow), the nuclei are stained blue with Dapi. (**B**). A representative section of the LV from Gal-3 wild-type IR mice shows increased Gal-3 expression in cardiac myocytes (thin arrow) around the infarction area, as well as in endothelial cells (arrowhead) and many neutrophil polymorphs in the infarction area (thick arrow). The nuclei are stained greenish blue because of increase expression of Gal-3 (green) in blue nuclei (Dapi). Immunofluorescent staining, Alexa Fluor 488.

Gal-3 levels were notably elevated in the left ventricle (LV) of Gal-3 wild-type mice 24 h after reperfusion (8870 ± 539.4 pg/mg protein) compared to sham-operated mice (4628 ± 345.1 pg/mg protein), *p* < 0.001, (Figure 2). Additionally, our immunofluorescent staining revealed a significant increase in Gal-3 expression in the LV section of the IR Gal-3 wild-type group when compared to the Gal-3 wild-type sham-operated mice (Figure 3).

### 3.3. Gal-3 Interacts with Autophagy Proteins

#### 3.3.1. Autophagy Flux

##### LC3B

**Figure 4:**
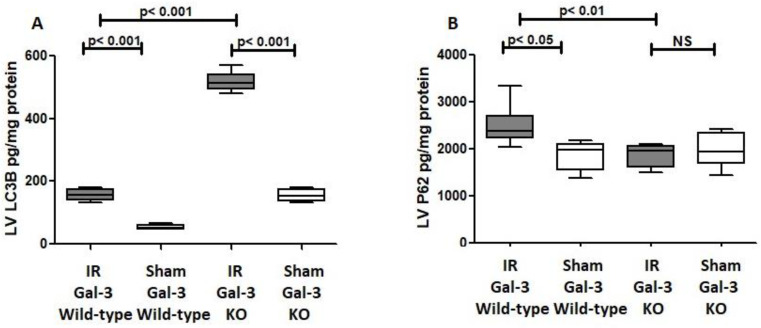
(**A**). The graph illustrates LV LC3B concentrations following IR in Gal-3 wild-type and IR Gal-3 KO mice, compared to their sham controls. (**B**). The graph shows LV p62 concentrations following IR in Gal-3 wild-type and IR Gal-3 KO mice, compared to their sham controls. A *p*-value of <0.05 is considered statistically significant.

**Figure 5:**
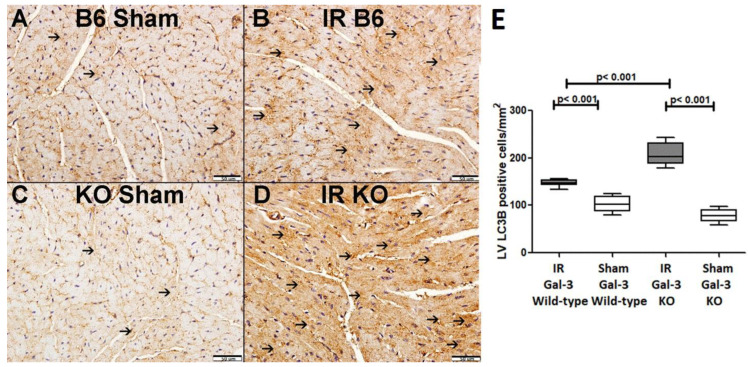
(**A**) representative section of the LV shows low cytoplasmic LC3B expression (thin arrow) in Gal-3 wild-type mice. (**B**). A representative section of the LV displays higher cytoplasmic LC3B expression (thin arrow) in Gal-3 wild-type IR mice. (**C**). A representative section of the LV shows low cytoplasmic LC3B expression (thin arrow) in Gal-3 KO sham mice. (**D**). A representative section of the LV exhibits higher cytoplasmic LC3B expression (thin arrow) in Gal-3 KO IR mice. (**E**). The graph illustrates LV morphometric analysis of LC3B cytoplasmic expression following IR in Gal-3 wild-type and IR Gal-3 KO mice, compared to their sham controls.

In the LVs of IR Gal-3 wild-type mice, LC3B concentrations were significantly elevated (156.4 ± 7.571 pg/mg protein) compared to Gal-3 wild-type sham control mice (53.29 ± 3.558 pg/mg protein) (*p* < 0.001) (Figure 4A). A similar increase in LC3B levels was observed in the LVs of IR Gal-3 KO mice (517.1 ± 12.83 pg/mg protein) compared to Gal-3 KO sham controls (154.2 ± 8.794 pg/mg protein) (*p* < 0.001) (Figure 4A). Notably, the LC3B concentration in the LVs of IR Gal-3 KO mice was considerably higher than in IR Gal-3 wild-type mice (*p* < 0.001) (Figure 4A). Statistical analysis using a one-way ANOVA followed by the Newman–Keuls post hoc test confirmed that these differences were significant (*p* < 0.001). Immunohistochemical staining shows higher expression of LC3B in LV cardiomyocytes in IR Gal-3 KO mice than in IR Gal-3 wild-type mice (Figure 5).

##### P62

**Figure 6:**
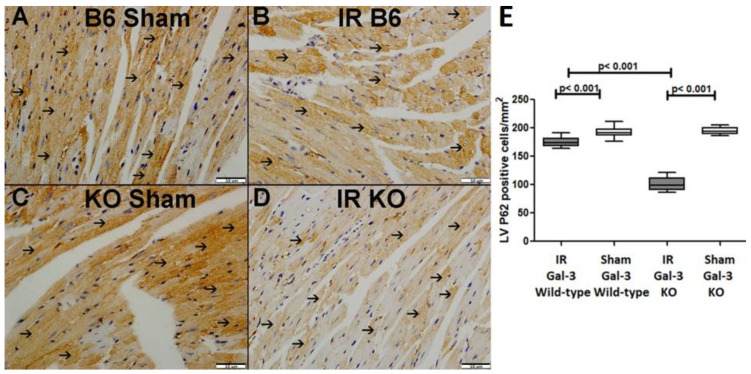
(**A**). A representative section of the LV shows cytoplasmic expression of p62 (thin arrow) in Gal-3 wild-type sham mice. (**B**). A representative section of the LV displays cytoplasmic expression of p62 (thin arrow) in Gal-3 wild-type IR mice. (**C**). A representative section of the LV shows cytoplasmic expression of p62 (thin arrow) in Gal-3 KO sham mice. (**D**). A representative section of the LV exhibits cytoplasmic expression of p62 (thin arrow) in Gal-3 KO IR mice. (**E**). The graph illustrates LV morphometric analysis of p62 cytoplasmic expression following IR in Gal-3 wild-type and IR Gal-3 KO mice, compared to their sham controls.

A significant increase in P62 concentration was observed in the LVs of ischemia–reperfusion (IR) Gal-3 wild-type mice (2512 ± 165.6 pg/mg protein) compared to Gal-3 wild-type sham control mice (1856 ± 139.7 pg/mg protein), indicating statistical significance (*p* < 0.05) (Figure 4B). In contrast, there was no significant increase in P62 levels in the LVs of IR Gal-3 KO mice (1872 ± 86.17 pg/mg protein) compared to Gal-3 KO control mice (1998 ± 165.1 pg/mg protein) (Figure 4B). Additionally, the P62 concentration in the LVs of IR Gal-3 wild-type mice was significantly higher than that in IR Gal-3 KO mice (*p* < 0.01) (Figure 4B). Immunohistochemical staining further confirmed that P62 expression was greater in LV cardiomyocytes of IR Gal-3 wild-type mice compared to IR Gal-3 KO mice (Figure 6).

##### Autophagy FLUX

The ELISA measurements revealed a significant increase in LV LC3B concentrations in both IR Gal-3 wild-type and IR Gal-3 KO mice 24 h after IR, indicating enhanced autophagy flux in response to myocardial injury. Since P62 is a key player in autophagy flux, higher autophagy flux typically correlates with lower P62 levels. Consequently, a reverse relationship between LC3B and P62 was observed. In IR Gal-3 KO mice, LC3B concentrations were higher, while P62 levels were lower in the LV compared to IR Gal-3 wild-type mice. This result suggests that the absence of Gal-3 is associated with increased autophagic flux, highlighting Gal-3′s potentially significant role in autophagy (Figure 4).

#### 3.3.2. p-AMPK

**Figure 7:**
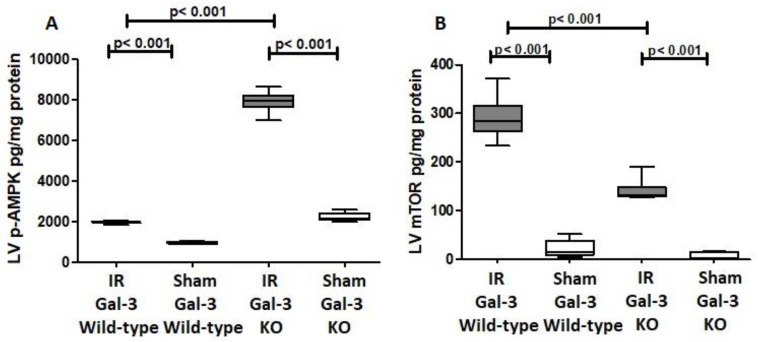
(**A**). The graph depicts left ventricular (LV) phosphorylated AMPK (p-AMPK) concentrations after ischemia–reperfusion (IR) in IR Gal-3 wild-type and IR KO mice, relative to their respective sham controls. (**B**). The graph illustrates LV phosphorylated mTOR (p-mTOR) concentrations following IR in IR Gal-3 wild-type and IR KO mice compared to their sham controls. A statistically significant difference is indicated by a *p* value < 0.05.

A significant increase in p-AMPK concentration was observed in the LVs of IR Gal-3 wild-type mice (1972 ± 27.84 pg/mg protein) compared to Gal-3 wild-type sham control mice (945.2 ± 32.03 pg/mg protein), with statistical significance (*p* < 0.001) (Figure 7A). Likewise, p-AMPK levels were significantly elevated in the LVs of IR Gal-3 KO mice (7912 ± 219.4 pg/mg protein) compared to Gal-3 KO sham control mice (2210 ± 101.5 pg/mg protein), also showing statistical significance (*p* < 0.001) (Figure 7A). Additionally, p-AMPK concentrations were significantly higher in the LVs of IR Gal-3 KO mice compared to IR Gal-3 wild-type mice (*p* < 0.001) (Figure 7A). Statistical analysis using one-way ANOVA followed by the Newman-Keuls post hoc test confirmed these differences were significant (*p* < 0.001).

**Figure 8:**
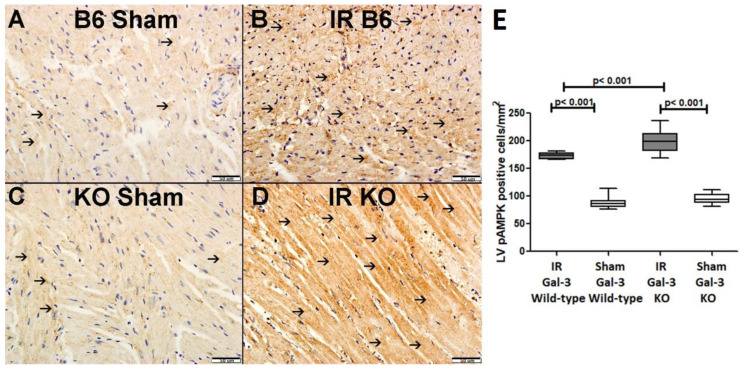
(**A**). A representative section of the LV showing low cytoplasmic expression of p-AMPK (thin arrow) in Gal-3 wild-type mice. (**B**). A representative section of the LV showing higher cytoplasmic expression of p-AMPK (thin arrow) in Gal-3 wild IR mice. (**C**). A representative section of the LV showing low cytoplasmic expression of p-AMPK (thin arrow) in Gal-3 KO sham mice. (**D**). A representative section of the LV showing higher cytoplasmic expression of p-AMPK (thin arrow) in Gal-3 KO IR mice. (**E**). The graph illustrates LV morphometric analysis of p-AMPK cytoplasmic expression following IR in Gal-3 wild-type and IR Gal-3 KO mice, compared to their sham controls.

Immunohistochemical staining shows higher expression of p-AMPK in LV cardiomyocytes in IR Gal-3 KO mice than in IR Gal-3 wild-type mice (Figure 8).

#### 3.3.3. p-mTOR

**Figure 9:**
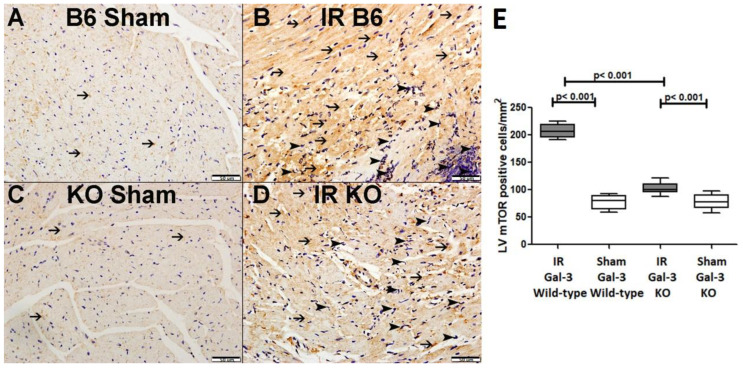
(**A**). A representative section of the LV shows cytoplasmic expression of p-mTOR in cardiomyocytes (thin arrow) in Gal-3 wild-type mice. (**B**). A representative section of the LV displays cytoplasmic expression of p-mTOR in cardiomyocytes (thin arrow) and neutrophil polymorphs (arrowhead) in Gal-3 wild-type IR mice. (**C**). A representative section of the LV shows cytoplasmic expression of p-mTOR in cardiomyocytes (thin arrow) in Gal-3 KO sham mice. (**D**). A representative section of the LV exhibits cytoplasmic expression of p-mTOR in cardiomyocytes (thin arrow) and neutrophil polymorphs (arrowhead) in Gal-3 KO IR mice. (**E**). The graph illustrates LV morphometric analysis of p-mTOR cytoplasmic expression following IR in Gal-3 wild-type and IR Gal-3 KO mice, compared to their sham controls.

A marked increase in the concentration of phosphorylated mammalian target of rapamycin (p-mTOR) was detected in the left ventricle (LV) of IR Gal-3 wild-type mice (290.5 ± 18.54 pg/mg protein) when compared to Gal-3 wild-type sham control mice (22.19 ± 8.051 pg/mg protein), demonstrating a statistically significant difference (*p* < 0.001) (Figure 7B). Likewise, p-mTOR levels were considerably elevated in the LVs of IR Gal-3 KO mice (140.9 ± 9.818 pg/mg protein) in comparison to Gal-3 KO sham controls (9.848 ± 3.120 pg/mg protein), also revealing statistical significance (*p* < 0.001) (Figure 7B). Furthermore, p-mTOR concentrations were significantly greater in the LVs of IR Gal-3 wild-type mice than in IR Gal-3 KO mice (*p* < 0.001) (Figure 7B). One-way ANOVA analysis followed by the Newman–Keuls post hoc test validated these differences as significant (*p* < 0.001). Additionally, immunohistochemical staining corroborated that p-mTOR expression was elevated in the LV cardiomyocytes of IR Gal-3 wild-type mice relative to those in IR Gal-3 KO mice (Figure 9).

#### 3.3.4. ULK-1

**Figure 10:**
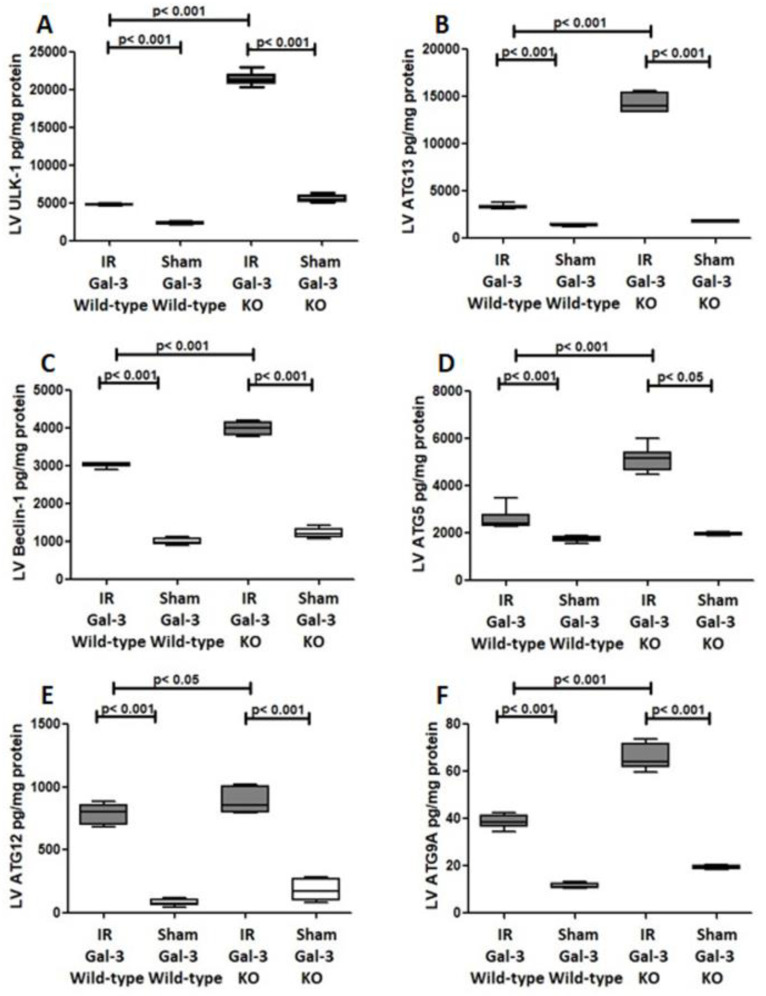
(**A**). The graph illustrates the concentrations of ULK-1 in the LV after IR in both IR Gal-3 wild-type and IR KO mice, compared to their respective sham control groups. (**B**). The graph displays the LV concentrations of ATG13 following IR in IR Gal-3 wild-type and IR KO mice, alongside their sham controls. (**C**). The graph shows LV Beclin-1 concentrations in IR Gal-3 wild-type and IR KO mice after IR, compared to their sham controls. (**D**). The graph depicts the levels of ATG5 in the LV following IR in IR Gal-3 wild-type and IR KO mice, in comparison to their sham controls. (**E**). The graph represents LV concentrations of ATG12 in IR Gal-3 wild-type and IR KO mice following IR, contrasted with their sham controls. (**F**). The graph highlights LV ATG9A concentrations in IR Gal-3 wild-type and IR KO mice after IR, relative to their sham control groups.

A notable increase in the concentration of ULK-1 was detected in the LVs of IR Gal-3 wild-type mice (1093 ± 20.11 pg/mg protein) when compared to Gal-3 wild-type sham control mice (653.9 ± 14.18 pg/mg protein), indicating statistical significance (*p* < 0.001) (see Figure 10A). Similarly, IR Gal-3 KO mice exhibited a significant rise in ULK-1 levels (1201 ± 36.09 pg/mg protein) compared to Gal-3 KO sham control mice (631.9 ± 10.12 pg/mg protein), also reflecting statistical significance (*p* < 0.001) (Figure 10A). Additionally, a significantly elevated concentration of ULK-1 was found in the LVs of IR Gal-3 KO mice relative to IR Gal-3 wild-type mice, with statistical significance (*p* < 0.001) (Figure 10A). Results from the multiple comparisons among the four experimental groups, assessed using a one-way ANOVA followed by the Newman–Keuls post hoc test, were determined to be statistically significant (*p* < 0.001).

#### 3.3.5. ATG13

A notable increase in the concentration of ATG13 was recorded in the LVs of IR Gal-3 wild-type mice (3287 ± 113.6 pg/mg protein) compared to Gal-3 wild-type sham control mice (1369 ± 41.75 pg/mg protein), indicating statistical significance (*p* < 0.001) (Figure 10B). Likewise, IR Gal-3 KO mice displayed a significant rise in ATG13 levels (14,280 ± 410.2 pg/mg protein) compared to Gal-3 KO sham control mice (1761 ± 52.15 pg/mg protein), also reflecting statistical significance (*p* < 0.001) (Figure 10B). Additionally, ATG13 concentrations were significantly higher in the LVs of IR Gal-3 KO mice than in IR Gal-3 wild-type mice, with statistical significance (*p* < 0.001) (Figure 10B). The results from multiple comparisons across the four experimental groups, assessed using a one-way ANOVA followed by the Newman–Keuls post hoc test, were confirmed to be statistically significant (*p* < 0.001).

#### 3.3.6. Beclin1

A significant increase in Beclin1 concentration was detected in the LVs of ischemia–reperfusion (IR) Gal-3 wild-type mice (3027 ± 27.48 pg/mg protein) compared to Gal-3 wild-type sham control mice (1004 ± 39.65 pg/mg protein), indicating statistical significance (*p* < 0.001) (Figure 10C). Similarly, a notable elevation in Beclin1 levels was observed in the LVs of IR Gal-3 KO mice (3991 ± 69.27 pg/mg protein) in comparison to Gal-3 KO sham control mice (1211 ± 58.94 pg/mg protein), also showing statistical significance (*p* < 0.001) (Figure 10C). Additionally, Beclin1 concentrations were significantly higher in the LVs of IR Gal-3 KO mice than in IR Gal-3 wild-type mice, with statistical significance (*p* < 0.001) (Figure 10C). The findings from multiple comparisons among the four experimental groups, analyzed using a one-way ANOVA followed by the Newman–Keuls post hoc test, confirmed these differences to be statistically significant (*p* < 0.001).

#### 3.3.7. ATG5

A significant increase in ATG5 concentrations was found in the LVs of IR Gal-3 wild-type mice (2573 ± 188.3 pg/mg protein) compared to Gal-3 wild-type sham control mice (1760 ± 51.07 pg/mg protein), indicating statistical significance (*p* < 0.001) Figure 10D). Similarly, a noteworthy elevation in ATG5 levels was observed in the LVs of IR Gal-3 KO mice (5122 ± 214.9 pg/mg protein) relative to Gal-3 KO sham control mice (1951 ± 28.81 pg/mg protein), which also demonstrated statistical significance (*p* < 0.05) (Figure 10D). Additionally, ATG5 concentrations were significantly higher in the LVs of IR Gal-3 KO mice compared to IR Gal-3 wild-type mice, with statistical significance (*p* < 0.001) (Figure 10D). The analysis of multiple comparisons among the four experimental groups, conducted using a one-way ANOVA followed by the Newman–Keuls post hoc test, confirmed these differences to be statistically significant (*p* < 0.001).

#### 3.3.8. ATG 12 

A significant increase in ATG12 concentrations was detected in the LVs of IR Gal-3 wild-type mice (788.8 ± 33.62 pg/mg protein) compared to Gal-3 wild-type sham control mice (81.74 ± 12.8 pg/mg protein), indicating statistical significance (*p* < 0.001) (see Figure 10E). Similarly, there was a notable elevation in ATG12 levels in the LVs of IR Gal-3 KO mice (888.2 ± 41.29 pg/mg protein) compared to Gal-3 KO sham control mice (183.9 ± 38.90 pg/mg protein), also demonstrating statistical significance (*p* < 0.001) (Figure 10E). Furthermore, ATG12 concentrations were significantly higher in the LVs of IR Gal-3 KO mice compared to IR Gal-3 wild-type mice, with statistical significance (*p* < 0.05) (Figure 10E). The analysis of multiple comparisons among the four experimental groups, performed using a one-way ANOVA followed by the Newman–Keuls post hoc test, confirmed these differences to be statistically significant (*p* < 0.001).

#### 3.3.9. ATG9A

A significant increase in ATG9A concentrations was observed in the LVs of IR Gal-3 wild-type mice (38.68 ± 1.290 pg/mg protein) compared to Gal-3 wild-type sham control mice (11.50 ± 0.5149 pg/mg protein), indicating statistical significance (*p* < 0.001) (Figure 10F). Similarly, a notable elevation in ATG9A levels was found in the LVs of IR Gal-3 KO mice (66.11 ± 2.468 pg/mg protein) relative to Gal-3 KO sham control mice (19.21 ± 0.3575 pg/mg protein), also demonstrating statistical significance (*p* < 0.001) (Figure 10F). Furthermore, ATG9A concentrations were significantly higher in the LVs of IR Gal-3 KO mice compared to IR Gal-3 wild-type mice, with statistical significance (*p* < 0.001) (Figure 10F). The results from multiple comparisons among the four experimental groups, assessed using a one-way ANOVA followed by the Newman–Keuls post hoc test, were confirmed to be statistically significant (*p* < 0.001).

### 3.4. GAL-3 Has an Antioxidant Effect in IR Injury

#### 3.4.1. GSH

**Figure 11:**
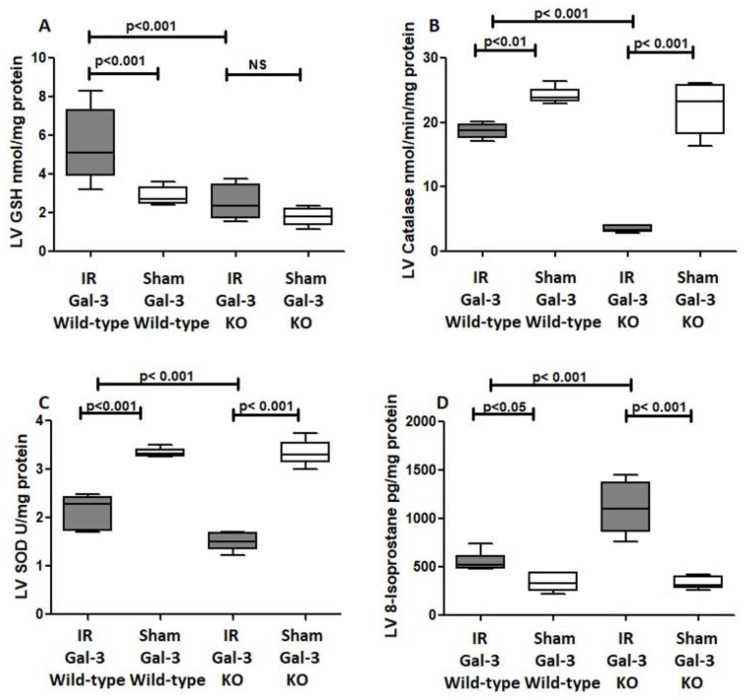
The graphs represent (**A**) left ventricular total GSH concentrations in the IR Gal-3 wild-type group and IR Gal-3 KO group compared to their sham controls. (**B**) Left ventricular catalase concentrations in the IR Gal-3 wild-type group and IR Gal-3 KO group compared to their sham controls. (**C**) Left ventricular SOD inhibition activity in the IR Gal-3 wild-type group and IR Gal-3 KO group compared to their sham controls. (**D**) Left ventricular 8-isoprostane concentrations in the IR Gal-3 wild-type group and IR Gal-3 KO group compared to their sham controls.

A significant increase in GSH concentrations was evident in the LVs of IR Gal-3 wild-type mice when (5.464 ± 0.7628 nmol/mg protein) compared with Gal-3 wild-type sham control mice (2.861 ± 0.1892 nmol/mg protein), signifying statistical significance (*p* < 0.001) (Figure 11A). Meanwhile, there was no significant elevation in GSH concentrations in the LV sof IR Gal-3 KO mice compared (2.515 ± 0.3594 nmol/mg protein) to Gal-3 KO sham control (1.801 ± 0.1917 nmol/mg protein) mice, (Figure 11A). Moreover, a significantly higher concentration of GSH was identified in the LVs of IR Gal-3 wild-type mice compared to IR Gal-3 KO mice, with statistical significance (*p* < 0.001) (Figure 11A). The results of multiple comparisons among the four experimental groups, utilizing a one-way ANOVA followed by the Newman–Keuls post hoc test, were found to be statistically significant (*p* < 0.001).

#### 3.4.2. Catalase

**Figure 12:**
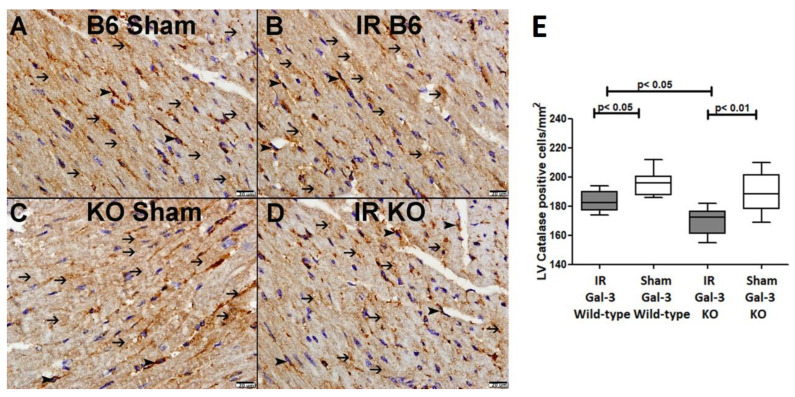
(**A**). A representative section of the LV showing cytoplasmic expression of catalase in cardiomyocytes (thin arrow) and endothelial cells (arrowhead) of Gal-3 wild-type sham mice. (**B**). A representative section of the LV showing cytoplasmic expression of catalase in cardiomyocytes (thin arrow) and endothelial cells (arrowhead) of Gal-3 wild-type IR mice. (**C**). A representative section of the LV showing cytoplasmic expression of catalase in cardiomyocytes (thin arrow) and endothelial cells (arrowhead) of Gal-3 KO sham mice. (**D**). A representative section of the LV showing cytoplasmic expression of catalase in cardiomyocytes (thin arrow) and endothelial cells (arrowhead) of Gal-3 KO IR mice. (**E**). The graph illustrates LV morphometric analysis of catalase cytoplasmic expression following IR in Gal-3 wild-type and IR Gal-3 KO mice, compared to their sham controls.

A significantly higher catalase concentration in the LVs of Gal-3 wild-type sham control mice (24.21 ± 0.509 nmol/min/mL) than IR Gal-3 wild-type mice (18.66 ± 0.4420 nmol/min/mL) was observed, signifying statistical significance (*p* < 0.01) (Figure 11B). There was a significantly higher catalase concentration in the LVs of Gal-3 KO sham control mice (22.30 ± 1.608 nmol/min/mL) than IR Gal-3 KO mice (3.475 ± 0.2004 nmol/min/mL), signifying statistical significance (*p* < 0.001) (Figure 11B). Moreover, a significantly higher concentration of catalase was identified in the LVs of IR Gal-3 wild-type mice compared to IR Gal-3 KO mice, with statistical significance (*p* < 0.001) (Figure 11B). The results of multiple comparisons among the four experimental groups, utilizing a one-way ANOVA followed by the Newman–Keuls post hoc test, were found to be statistically significant (*p* < 0.001). Immunohistochemical staining further verified that catalase expression was greater in LV cardiomyocytes of IR Gal-3 wild-type mice compared to IR Gal-3 KO mice (Figure 12).

#### 3.4.3. SOD

A significant higher SOD concentration in the LVs of Gal-3 wild-type sham control mice (3.344 ± 0.03520 U/mg protein) than IR Gal-3 wild-type mice (2.150 ± 0.1408 U/mg protein) was observed, signifying statistical significance (*p* < 0.001) (Figure 11C). There was a significantly higher SOD concentration in the LVs of Gal-3 KO sham control mice (3.339 ± 0.1052 U/mg protein) than in IR Gal-3 KO mice (1.500 ± 0.0737 U/mg protein), signifying statistical significance (*p* < 0.001) (Figure 11C). Moreover, a significantly higher concentration of SOD was identified in the LVs of IR Gal-3 wild-type mice compared to IR Gal-3 KO mice, with statistical significance (*p* < 0.001) (Figure 11C). The results of multiple comparisons among the four experimental groups, utilizing a one-way ANOVA followed by the Newman–Keuls post hoc test, were found to be statistically significant (*p* < 0.001).

#### 3.4.4. 8-Isoproatane

A significantly higher 8-isoprostane concentration in the LVs of IR Gal-3 wild-type mice (553.7 ± 39.65 pg/mg protein) than Gal-3 wild-type sham control mice (337.2 ± 37.33 pg/mg protein) was observed, signifying statistical significance (*p* < 0.05) (Figure 11D). There was a significantly higher 8-isoprostane concentration in the LVs of IR Gal-3 KO mice (1107 ± 113.9 pg/mg protein) than in Gal-3 KO sham control mice (328.2 ± 26.04 pg/mg protein), signifying statistical significance (*p* < 0.001) (Figure 11C,D). Moreover, a significantly higher concentration of 8-isoprostane was identified in the LVs of IR Gal-3 KO mice compared to IR Gal-3 wild-type mice, with statistical significance (*p* < 0.001) (Figure 11D). The results of multiple comparisons among the four experimental groups, utilizing a one-way ANOVA followed by the Newman–Keuls post hoc test, were found to be statistically significant (*p* < 0.001).

#### 3.4.5. NRF2

**Figure 13:**
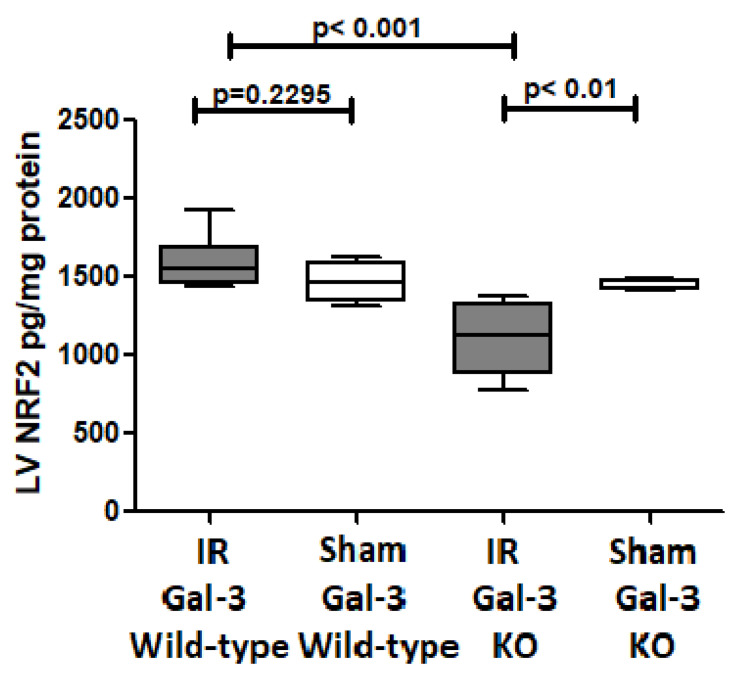
The graphs represent left ventricular NRF2 concentrations in IR Gal-3 wild-type mice and Gal-3 KO IR group compared to their sham controls.

**Figure 14:**
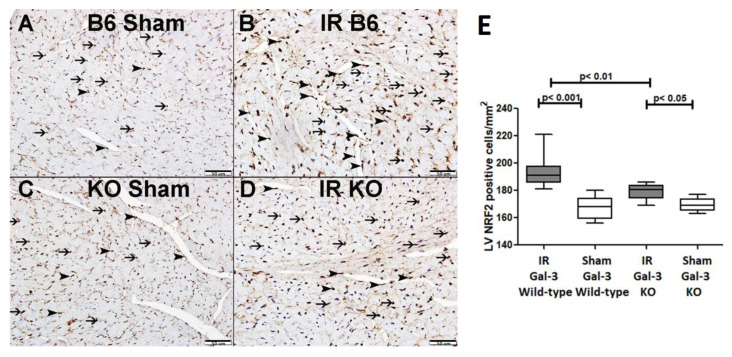
(**A**). A representative section of the LV showing nuclear expression of catalase in cardiomyocytes (thin arrow) and endothelial cells (arrowhead) of Gal-3 wild-type sham mice. (**B**). A representative section of the LV showing nuclear expression of catalase in cardiomyocytes (thin arrow) and endothelial cells (arrowhead) of Gal-3 wild-type IR mice. (**C**). A representative section of the LV showing nuclear expression of catalase in cardiomyocytes (thin arrow) and endothelial cells (arrowhead) of Gal-3 KO sham mice. (**D**). A representative section of the LV showing nuclear expression of catalase in cardiomyocytes (thin arrow) and endothelial cells (arrowhead) of Gal-3 KO IR mice. (**E**). The graph illustrates LV morphometric analysis of NRF2 nuclear expression following IR in Gal-3 wild-type and IR Gal-3 KO mice, compared to their sham controls.

There was no significant difference in NRF2 concentrations between the LVs of IR Gal-3 wild-type mice (1531 ± 73.13 pg/mg protein) and Gal-3 wild-type sham control mice (1467 ± 58.02 pg/mg protein), signifying statistical significance (*p* = 0.2295) (Figure 13). There was a significantly higher NRF2 concentration in the LVs of Gal-3 KO sham mice (1442 ± 13.92 pg/mg protein) than IR Gal-3 KO mice (1107 ± 93.83 pg/mg protein), signifying statistical significance (*p* < 0.01) (Figure 13). Moreover, a significantly higher concentration of NRF2 was identified in the LVs of IR Gal-3 wild-type mice compared to IR Gal-3 KO mice, with statistical significance (*p* < 0.001) (Figure 13). The results of multiple comparisons among the four experimental groups, utilizing a one-way ANOVA followed by the Newman–Keuls post hoc test, were found to be statistically significant (*p* < 0.001). Immunohistochemical staining further verified that the NRF2 expression was greater in the LV cardiomyocytes of IR Gal-3 wild-type mice compared to IR Gal-3 KO mice (Figure 14).

#### 3.4.6. H_2_O_2_

**Figure 15:**
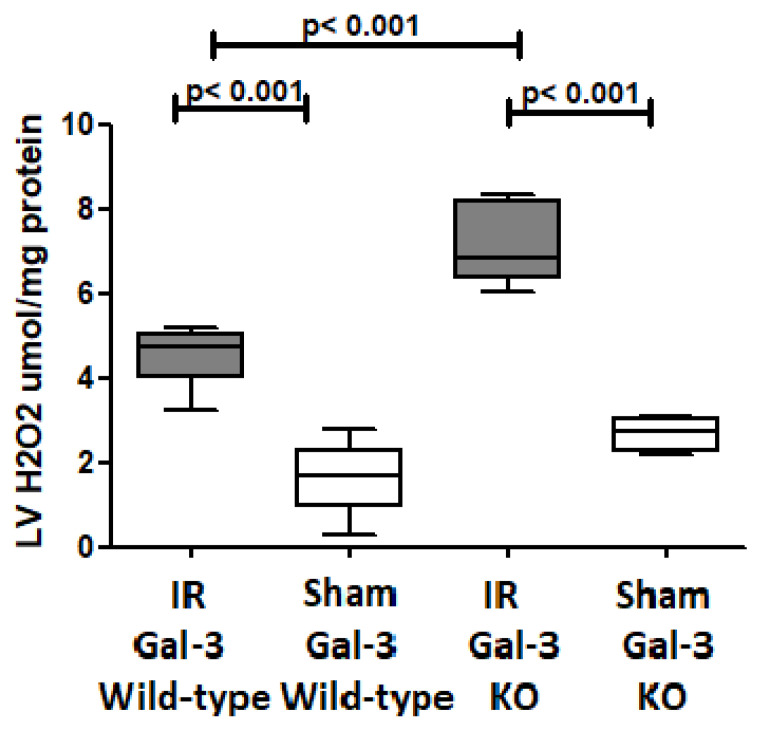
The graphs represent left ventricular H_2_O_2_ concentrations in IR Gal-3 wild-type mice and Gal-3 KO IR group compared to their sham controls.

A significantly higher H_2_O_2_ concentration was detected in the LVs of IR Gal-3 wild-type mice (4.554 ± 0.2885 pg/mg protein) than Gal-3 wild-type sham control mice (1.646 ± 0.3978 pg/mg protein), signifying statistical significance (*p* < 0.001) (Figure 15). There was a significantly higher H_2_O_2_ concentration in the LVs of IR Gal-3 KO mice (7.126 ± 0.3796 pg/mg protein) than Gal-3 KO sham control mice (2.698 ± 0.1566 pg/mg protein), signifying statistical significance (*p* < 0.001) (Figure 15). Moreover, a significantly higher concentration of H_2_O_2_ was identified in the LVs of IR Gal-3 KO mice compared to IR Gal-3 wild-type mice, with statistical significance (*p* < 0.001) (Figure 15). The results of multiple comparisons among the four experimental groups, utilizing a one-way ANOVA followed by the Newman–Keuls post hoc test, were found to be statistically significant (*p* < 0.001).

## 4. Discussion

Ischemia–reperfusion (IR) injury in the heart is a topic of intense interest since thrombolytic therapy and percutaneous coronary intervention became the mainstay of treatment for patients with acute myocardial infarction. Liu Y et al. showed that the mechanism underlying IR injury are multifactorial and involve a variety of processes, including oxidative stress and inflammation leading to cellular damage in the form of necrosis, apoptosis and autophagy [35]. During the ischemic period, there is a reduction in oxygen and nutrient supply, leading to a decrease in ATP production and an accumulation of metabolic waste products, then, when blood flow is restored, a sudden increase in oxygen and nutrient supply can lead to the generation of reactive oxygen species (ROS), which can cause oxidative damage to cellular structures, such as lipids, proteins, and DNA, leading to apoptosis, necrosis, and autophagy [36].

We showed that plasma troponin I concentrations are significantly higher in IR Gal-3 wild-type and IR Gal-3 KO mice than in corresponding sham control mice, confirming LV cardiomyocyte necrosis at 24 h following reperfusion. We have also identified that plasma troponin I concentrations are higher in IR Gal-3 KO mice than in GAL-3 wild-type mice suggesting higher cardiomyocyte death in IR Gal-3 KO mice than in IR Gal-3 wild-type mice. This finding supports the idea that the absence of intracellular Gal-3 may lead to increased cardiomyocyte death in ischemic myocardium during IR injury [37].

Additionally, we showed a significant increase in LV Gal-3 concentration in Gal-3 wild IR mice compared to their sham control group at 24 h following reperfusion, and immunofluorescent staining of LVs has also shown a significantly higher expression of intracellular cytoplasmic and nuclear Gal-3 in peri-infarction cardiomyocytes, endothelial cells, and neutrophil polymorphs, suggesting the important role of Gal-3 at early IR injury. These findings are compatible with our previous studies [36,37,38]. Autophagy is an intracellular mechanism involving the breakdown of damaged proteins and organelles through lysosomes, repurposing the resulting components for various cellular functions, and then, during IR injury, autophagy triggers energy production, recycles damaged proteins and organelles, and eliminates intracellular toxins [7,39]. We observed a notable rise in autophagy flux and autophagy-related proteins in both IR Gal-3 wild-type mice and IR Gal-3 KO mice, indicating enhanced autophagy during myocardial ischemia–reperfusion injury. We also demonstrated that IR Gal-3 KO mice exhibit higher LV autophagy flux and autophagy-related proteins compared to IR Gal-3 wild-type mice, suggesting that the loss of intracellular Gal-3 is associated with increased autophagy.

Changes in autophagic proteins like LC3B and p62 further illuminate Gal-3′s role in regulating autophagy. Weng IC et al. have also shown that the loss of intracellular Gal-3 results in enhanced autophagy activation through increased cytoplasmic LC3B, and reciprocally, increased intracellular Gal-3 can create strong anti-autophagic activity [32].

The marked rise in LC3B levels in the LVs of IR mice groups, especially in Gal-3 KO mice, indicates an elevated autophagic flux. LC3B’s crucial function in autophagy (encompassing vesicle elongation, maturation, autophagosome–lysosome fusion, and cargo recognition) supports the conclusion of increased autophagy flux. Both Debnath J et al. [40] and Lee YK et al. [41] support our conclusion that increased LC3B indicates increased autophagic flux [40,41].

Furthermore, the significantly lower concentrations of p62 in the LVs of IR Gal-3 KO mice compared to IR Gal-3 wild-type mice support the notion of increased autophagic flux. As p62 is typically degraded during autophagy, its reduced levels in IR Gal-3 KO mice suggest enhanced autophagic turnover. This finding is consistent with our previous study on autophagy in renal tubules during acute tubular necrosis, which also demonstrated that decreased p62 levels correlate with increased autophagy [42]. Additionally, our results reveal a significant increase in the concentrations of various autophagy-related proteins, including ULK1, ATG13, Beclin-1, ATG5, ATG12, and ATG9A, in the LV affected by myocardial IR injury when compared to sham controls. This finding suggests that autophagy is activated in response to IR injury, supporting the idea that autophagy is often induced under stress conditions, such as oxidative stress. Furthermore, the significantly higher concentrations of these autophagy proteins in IR Gal-3 KO mice compared to IR Gal-3 wild-type mice indicate that the absence of the Gal-3 gene may enhance autophagy.

We also observed notably higher levels of phosphorylated AMP-activated protein kinase (p-AMPK) in the left ventricles of IR Gal-3 knockout mice compared to their IR Gal-3 wild-type counterparts. This finding suggests that the absence of the Gal-3 gene might enhance autophagy, a process in which p-AMPK is a critical regulator. AMPK promotes autophagy by directly phosphorylating Ulk-1 at several sites, including Ser467, Ser555, Thr574, and Ser637, which enhances Ulk-1 activity. This activity aligns with our observation of elevated LV p-AMPK and Ulk-1 levels during IR injury and is consistent with increased autophagy flux [43]. This action boosts the transcription and recruitment of proteins necessary for autophagy to specific membrane domains, initiating autophagosome formation and eventually leading to autolysosome development [43,44].

The elevated levels of p-AMPK in IR Gal-3 knockout mice further indicate that Gal-3 might function as a negative regulator of AMPK-driven autophagy, which is supported by the lower concentrations of p-mTOR (phosphorylated mammalian target of rapamycin) observed in the LVs of IR Gal-3 knockout mice compared to their wild-type counterparts. Mizushima N et al. showed how mTOR suppresses autophagy, which occurs by phosphorylating Ulk-1 and ATG13 and prevents the formation of the Ulk-1-Ulk2 complex, thereby inhibiting the initiation of autophagy [45].

The intricate relationship among AMPK, mTOR, and autophagy is well established. Phosphorylated AMPK (p-AMPK) can directly stimulate autophagy by phosphorylating ULK1, and it also inhibits mTOR via phosphorylation, thereby indirectly promoting autophagy [44]. In the context of our study, the elevated levels of p-AMPK and reduced levels of p-mTOR in Gal-3 KO mice suggest that the absence of the Gal-3 gene may enhance autophagy by increasing p-AMPK and inhibiting mTOR. The reciprocal regulation between AMPK and mTOR—where AMPK inhibits the activity of the mTOR complex 1 (TORC1), and, in turn, TORC1 suppresses AMPK activation—further supports the notion that the loss of Gal-3 disrupts this balance in favor of increased autophagy [46]. Zing et al. demonstrated that elevated intracellular Gal-3 levels can inhibit autophagy through the activation of mTORC1 [47]. Our findings provide insights into the molecular mechanisms by which Gal-3 may modulate autophagy, highlighting the complex interactions between AMPK and mTOR. A deeper understanding of these regulatory pathways could pave the way for targeted interventions in conditions characterized by dysregulated autophagy, such as myocardial ischemia–reperfusion injury.

During IR injury, there is an increase in oxidative stress due to the generation of reactive oxygen species (ROS) [48]. Our study showed an increase in oxidative stress during IR injury (Figure 11) with significantly higher LV 8-isoprostane and H_2_O_2_ concentrations in both IR Gal-3 wild-type and IR Gal-3 KO mice than their sham controls. Additionally, there is significantly higher LV 8-isoprostane and H_2_O_2_ concentrations in IR Gal-3 KO mice than in IR Gal-3 wild-type mice, supporting the notion that the loss of Gal-3 is associated with higher oxidative stress. On the other hand, there is significantly higher antioxidant activities in the LVs of IR Gal-3 wild-type mice than in IR Gal-3 KO mice (Figure 11A–C) supporting the idea that increased LV Gal-3 is associated with increased antioxidant activity [35]. Our observations of increased autophagy in the LV of IR injury are linked to increased oxidative stress at 24 h following IR injury, suggesting that oxidative stress can trigger autophagy [49,50].

ROS are generated from mitochondria. Then, the accumulation of ROS oxidizes and damages cellular proteins, DNA, and lipids and stimulates autophagy to degrade damaged intracellular components to be then reutilized by injury cells [51]. Mitochondrial H_2_O_2_ has important roles in cellular signaling, which is rather stable compared to the other ROS molecules, and can easily diffuse to cytosol [52]. In response to nutrient deprivation during cell injury, H_2_O_2_ enables the reduced form of ATG4 to convert LC3B-I to LC3B-II through thiol modification of the Cys81 of ATG4, leading to increased autophagosome formation [53]. Zhang et al. showed that the treatment of malignant glioma cell lines with H_2_O_2_ stimulates both autophagy and apoptosis in treated cells [54]. H_2_O_2_ directly activates AMPK by oxidizing cysteine residues of α and β subunits [55]. Moreover, H_2_O_2_ activates AMPK through phosphorylation at threonine 172 (T172) by liver kinase B1 (LKB1), which suppresses mTORC1, and, thus, induces autophagy [56]. Our study also showed a significantly higher concentration of H_2_O_2_ in the LVs of IR mice groups than their corresponding sham controls, supporting increased autophagy flux during IR injury. Our finding is supported by Matarrese P et al., who also showed that increased intracellular Gal-3 can decrease mitochondrial H_2_O_2_ and protect cells from being damaged by ROS [57]. Our observation of higher H_2_O_2_ concentrations in IR Gal-3 KO mice than in Gal-3 wild-type mice correlates well with higher autophagy flux in IR Gal-3 KO mice than in Gal-3 wild-type mice through cross talk between Gal-3, ROS, and autophagy.

Additionally, studies have shown that the interaction between mitochondrial ROS and Ca^2+^ signaling plays a significant role in regulating autophagy because under hypoxic conditions, mitochondrial ROS facilitate the movement of stromal interaction molecule 1 (STIM1) to the plasma membrane, where it activates Ca^2+^ release-activated Ca^2+^ (CRAC) channels, and, then, this activation leads to an increase in Ca^2+^ influx, triggering calcium/calmodulin-dependent protein kinase kinase 2 (CAMKK2), which then activates AMPK and promotes autophagy [58]. Furthermore, mitochondrial ROS also activate the lysosomal Ca^2+^ channel mucolipin-1 (MCOLN1), causing Ca^2+^ release and the calcineurin-dependent nuclear translocation of transcription factor EB (TFEB), which results in the transcription of genes related to autophagy and lysosomal function [59].

Additionally, our results indicate elevated levels of nuclear factor erythroid 2-related factor 2 (NRF2) in the LVs of IR mice, with significantly higher levels observed in IR Gal-3 wild-type mice compared to IR Gal-3 KO mice. NRF2 serves as a key transcription factor that regulates the expression of various genes encoding antioxidant and detoxifying enzymes, which are essential for maintaining cellular redox homeostasis [60]. Kelch-like ECH-associated protein 1 (KEAP1) functions as a substrate adaptor protein within a larger E3 ubiquitin ligase complex, facilitating the ubiquitination and proteasomal degradation of NRF2 [61]. In response to oxidative stress, NRF2 dissociates from KEAP1 and translocates to the nucleus, where it binds to an antioxidant response element (ARE) sequence to activate its target genes [60]. In the context of autophagic signaling, NRF2 enhances p62 gene expression in response to oxidative stress, and the resulting protein further activates NRF2, creating a positive feedback loop [62]. Gal-3 can modulate NRF2 activity by activating the PI3K/AKT pathway, leading to the phosphorylation and inactivation of glycogen synthase kinase 3 beta (GSK-3β), which negatively regulates NRF2. Consequently, an increase in intracellular Gal-3 enhances NRF2 stability and promotes its nuclear translocation, resulting in the increased transcription of antioxidant genes [63,64,65,66].

Trimarchi G et al. showed that transient ventricular dysfunction (TVF) can occur following myocardial IR [67]. They showed molecular changes associated with TVF, including metabolism shift, cellular dedifferentiation, oxidative stress, apoptosis, and fibrosis [67]. It was also shown that ischemic preconditioning (IPC) and post conditioning (IPostC) can protect cardiomyocytes during IR injury; furthermore, the molecular mechanisms that occur during IPC and IPostC are not only protecting cardiomyocytes from ischemia–reperfusion injury but also modulate autophagy [68]. During preconditioning, brief ischemic episodes activate signaling pathways like AMPK and PI3K/Akt, which promote a controlled autophagic response, which helps clear damaged proteins and organelles, maintaining cellular homeostasis and reducing stress during the subsequent ischemic event [68]. In postconditioning, the inhibition of excessive autophagy is critical through the activation of the reperfusion injury salvage kinase pathway and the survivor activating factor pathway during early reperfusion prevents the hyperactivation of autophagy, which may lead to cell death [68]. By finely regulating autophagy, IPC and IPostC enhance cardiomyocyte survival and prevent the excessive degradation of cellular components, thus improving recovery from ischemic injury [67,68].

As previously discussed, we believe that there are cross talks between Gal-3, ROS, NRF2, p-AMPK, mTOR, and autophagy-related proteins. An increase in intracellular Gal-3 may help maintain mitochondrial homeostasis by stabilizing mitochondrial membranes, which leads to the reduced production of ROS and H_2_O_2_, eventually resulting in reduction in autophagy. Additionally, high intracellular Gal-3 promotes the translocation of NRF2 to the nucleus, where it binds to ARE and enhances the transcription of antioxidant genes, ultimately resulting in the downregulation of autophagy. Additionally, Gal-3 appears to inhibit autophagy by activating mTORC1 and suppressing p-AMPK.

## 5. Conclusions

Our study supports the interaction of Gal-3 with autophagy proteins, ROS, and antioxidant proteins and demonstrates that the absence of Gal-3 can enhance autophagy in the heart during IR injury.

## Data Availability

The data presented in this study are available upon request from the corresponding author.
